# Mad River Valley Dental Association

**Published:** 1865

**Authors:** J. Blake


EDITOR REGISTER: The dentists of the Mad River Valley met in the city of Springfield on the 27th of April, for the purpose of reorganizing the Mad River Valley Dental Association.

The attendance was large for the limited notice given, and all expressed a determination that the reorganization should be effected upon a basis which would insure its future success.

A temporary organization was effected, by appointing Dr. G. L. PAINE President, Dr. GEORGE WATT Corresponding Secretary, and Dr. J. BLAKE Recording Secretary.

A committee of three was appointed to draft a constitution and by-laws; also, a committee of three to select subjects for discussion and essayists for next meeting.

The subjects selected were as follows:

1st. Filling Teeth—the best mode and material.

2d. The comparative value of the different kinds of plate work.

Dr. BLOUNT, the President under the old organization, read an essay, which was requested for publication.

After considerable discussion upon various subjects connected with the profession, the Society adjourned, to meet in Urbana on the first Tuesday in June, at which time a full attendance is expected. J. BLAKE, Sec.

